# Müller glial dysfunction during diabetic retinopathy in rats is reduced by the acrolein-scavenging drug, 2-hydrazino-4,6-dimethylpyrimidine

**DOI:** 10.1007/s00125-018-4707-y

**Published:** 2018-08-15

**Authors:** Rosemary E. McDowell, Peter Barabas, Josy Augustine, Olivier Chevallier, Philip McCarron, Mei Chen, J. Graham McGeown, Tim M. Curtis

**Affiliations:** 1 Centre for Experimental Medicine, School of Medicine, Dentistry & Biomedical Science, Queen’s University Belfast, 97 Lisburn Road, Belfast, BT9 7BL UK; 2 Advanced Mass Spectrometry Core Technology Unit, Faculty of Medicine, Health and Life Sciences, Queen’s University Belfast, Belfast, UK

**Keywords:** Acrolein, Advanced lipoxidation end-products, Diabetic retina, Electroretinography, Hydrazino compounds, Inflammatory signalling, Müller glia, Oxidative stress, Retinopathy, Scavenging agents

## Abstract

**Aims/hypothesis:**

Recent studies suggest that abnormal function in Müller glial cells plays an important role in the pathogenesis of diabetic retinopathy. This is associated with the selective accumulation of the acrolein-derived advanced lipoxidation end-product, *N*^ε^-(3-formyl-3,4-dehydropiperidino)lysine (FDP-lysine), on Müller cell proteins. The aim of the current study was to identify more efficacious acrolein-scavenging drugs and determine the effects of the most potent on Müller cell FDP-lysine accumulation and neuroretinal dysfunction during diabetes.

**Methods:**

An ELISA-based in vitro assay was optimised to compare the acrolein-scavenging abilities of a range of drugs. This identified 2-hydrazino-4,6-dimethylpyrimidine (2-HDP) as a new and potent acrolein scavenger. The ability of this agent to modify the development of diabetic retinopathy was tested in vivo. Male Sprague Dawley rats were divided into three groups: (1) non-diabetic; (2) streptozotocin-induced diabetic; and (3) diabetic treated with 2-HDP in their drinking water for the duration of diabetes. Liquid chromatography high-resolution mass spectrometry was used to detect 2-HDP reaction products in the retina. Immunohistochemistry, real-time quantitative (q)RT-PCR and electroretinography were used to assess retinal changes 3 months after diabetes induction.

**Results:**

2-HDP was the most potent of six acrolein-scavenging agents tested in vitro (*p* < 0.05). In vivo, administration of 2-HDP reduced Müller cell accumulation of FDP-lysine at 3 months in rats rendered diabetic with streptozotocin (*p* < 0.001). A 2-HDP adduct was identified in the retinas of diabetic animals treated with this compound. 2-HDP supplementation was associated with reduced Müller cell gliosis (*p* < 0.05), reduced expression of the oxidative stress marker haem oxygenase-1 (*p* < 0.001) and partial normalisation of inwardly rectifying K^+^ channel 4.1 (Kir4.1) expression (*p* < 0.001 for staining in perivascular regions and the innermost region of the ganglion cell layer). Diabetes-induced retinal expression of inflammatory markers, inflammatory signalling compounds and activation of retinal microglial cells were all reduced in 2-HDP-treated animals. Retinal neurophysiological defects in diabetic animals, as indicated by changes in the electroretinogram 7 weeks after induction of diabetes, were also reduced by 2-HDP (*p* < 0.05–0.01 for b-wave amplitudes at flash intensities from −10 to +10 dB; *p* < 0.01 for time to peak of summed oscillatory potentials at +10 dB).

**Conclusions/interpretation:**

These findings support the hypothesis that Müller cell accumulation of FDP-lysine plays an important role in the development of diabetic retinopathy. Our results also suggest that 2-HDP may have therapeutic potential for delaying or treating this sight-threatening complication.

**Electronic supplementary material:**

The online version of this article (10.1007/s00125-018-4707-y) contains peer-reviewed but unedited supplementary material, which is available to authorised users.

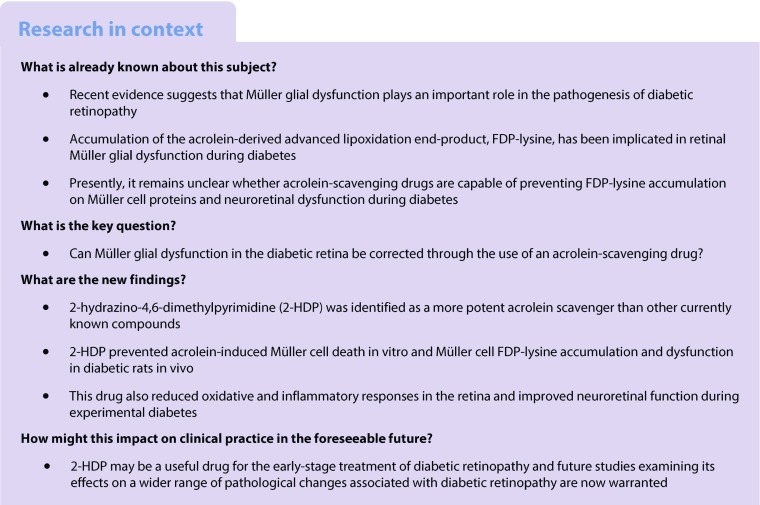



## Introduction

Diabetic retinopathy is a leading cause of newly diagnosed blindness in people of working age in industrialised nations [1]. Although there is no question that microvascular pathology is central to the development of this condition, in recent years it has become increasingly clear that diabetes also significantly impacts neuronal and glial cell function in the retina [2, 3]. Müller cells, which are the principal glial cells of the retina and span its entire thickness, are known to be particularly vulnerable to damage in diabetes. Positioned between the retinal neurons and blood vessels, these cells normally play a fundamental role in providing structural and neurotrophic support to the retina, regulating retinal metabolism, water, ion and neurotransmitter homeostasis and matching retinal blood flow to changes in neuronal activity (functional hyperaemia) [4]. In diabetes, many of these important functions become disturbed and the cells assume a reactive phenotype characterised by the upregulation of glial fibrillary acidic protein (GFAP) and the production of inflammatory chemokines and cytokines [5]. Given the importance of Müller cells in maintaining neurovascular structure and function in the retina, their dysfunction in diabetes is believed to be a major factor that contributes to the evolution of the microvascular and neurodegenerative lesions of diabetic retinopathy [6, 7].

While considerable progress has been achieved in defining the nature of Müller cell abnormalities during diabetic retinopathy, less is known about the exact biochemical and molecular mechanisms through which diabetes affects these cells. Recent evidence from our laboratory, however, has suggested that accumulation of the acrolein (ACR)-derived protein adduct, *N*^ε^-(3-formyl-3,4-dehydropiperidino)lysine (FDP-lysine), on Müller cell proteins may play a significant role [8, 9]. Selective accumulation of FDP-lysine is observed in Müller cells within just a few months of experimental diabetes and, when modelled in vitro, initiates Müller cell dysfunction consistent with that observed in the diabetic retina, including the induction of oxidative stress, generation of inflammatory mediators and disruption of ionic homeostatic mechanisms [9]. We have also reported that when administered at high concentrations, the carbonyl-scavenging drug, pyridoxamine, is moderately effective at reducing FDP-lysine formation and Müller cell dysfunction in diabetic rodents [8]. However, we have also found that pyridoxamine has deleterious effects on the electroretinogram (ERG) in diabetic animals [10], raising concerns about the potential usefulness of this drug for the long-term treatment of diabetic retinopathy.

It is evident from these previous studies that targeting Müller cell ACR/FDP-lysine accumulation could provide new avenues for preventing neurovascular pathology during diabetic retinopathy. Drugs capable of efficiently blocking their formation in the absence of any adverse effects have yet to be identified. 2-Mercaptoethane sulfonate (MESNA) is an extremely potent thiol-based scavenger of ACR that is used as an adjunctive therapy, along with alkylating chemotherapy drugs [11]. Although effective as a treatment for acute ACR toxicity resulting from the metabolism of these agents, the hydrophilicity of MESNA prevents it from readily entering cells, making it unsuitable for the routine sequestering of Müller cell ACR within the eye [12]. Besides MESNA, the hydrazino compound hydralazine (HDZ) has been identified as an effective ACR scavenger [13]. HDZ has been approved clinically as an anti-hypertensive drug for many years [14], but its cardiovascular actions complicate its potential use for the treatment of diabetic retinopathy. As it is the hydrazine groups on hydralazine that are known to be responsible for its ACR-scavenging abilities [15] and not all hydrazine-containing compounds lower blood pressure [16], other compounds containing hydrazino groups could have therapeutic value for preventing Müller cell ACR/FDP-lysine accumulation and dysfunction in diabetes.

In the present study, we screened a small panel of hydrazino and other nucleophilic compounds for their ability to scavenge ACR and compared our results with those obtained using MESNA and HDZ. Subsequently, we tested the effects of the most potent compound, 2-hydrazino-4,6-dimethylpyrimidine (2-HDP), on Müller cell FDP-lysine accumulation and neuroretinal dysfunction in diabetic rodents.

## Methods

### Diabetic rat model

All experiments were conducted in accordance with the National Institutes of Health (NIH) Guide for the Use and Care of Laboratory Animals (8th edition, 2011) and approved by the Queen’s University of Belfast Animal Welfare and Ethical Review Body. Work adhered to Department of Health, Social Services and Public Safety (DHSSPS)/Home Office project licence PPL2654.

Animals were assigned to experimental groups by simple randomisation. Male Sprague Dawley rats (7–8 weeks of age; ~200 g body weight; Harlan, Bicester, UK) were rendered diabetic by a single intraperitoneal injection of streptozotocin (STZ; Sigma-Aldrich, Poole, UK; 65 mg/kg in 0.1 mol/l citrate buffer, pH 4.6). Citrate-buffer-injected age-matched rats were used as the control group. One week after STZ injection, blood glucose measurements were made by glucometric assessment of tail-prick blood samples (Breeze2, Bayer Scientific, Leverkusen, Germany). Animals with blood glucose concentrations >15 mmol/l were used in the study and a subset of these diabetic animals received the ACR-scavenging drug, 2-HDP (synthesised to >97% purity by Fartop, Nanjing, China) administered orally in their drinking water (dissolved directly at 100 mg/l). All animals were housed in standard cages under a 12 h light–dark cycle in the Biological Services Unit (BSU) at Queen’s University Belfast with free access to food and water. Animal weights and water consumption were monitored daily. At 3 months’ diabetes duration, rats were euthanised by CO_2_ asphyxiation and blood collected via cardiac puncture for measurement of HbA_1c_ levels (GLYCO-Tek kit, Helena Biosciences Europe, Gateshead, UK). No adverse events were associated with the 2-HDP intervention. No specific blinding was carried out to outcome assessment.

### ELISA for drug scavenging of ACR

We developed an ELISA assay to assess the ability of a small panel of hydrazino and other nucleophilic compounds to scavenge ACR and prevent FDP-lysine adduct formation on the model protein, human serum albumin (HSA). The selected compounds had no known cardiovascular actions [16] but retained drug-like physiochemical properties (according to Lipinski’s rule of five) [17]. The known ACR-scavenging drugs, HDZ and MESNA, were included for comparison. Chemicals were purchased from the following companies: HDZ, MESNA, 1,2-diphenylhydrazine (DPH) and 2-aminopropanol (2-AP) from Sigma-Aldrich; 2-HDP from Chembridge Corporation (San Diego, CA, USA) and 2-hydrazinoquinoline (2-HQ) from Fluorochem (Hadfield, UK). Full details of the ELISA are provided in the electronic supplementary material (ESM) Methods.

### Müller cell viability

The RealTime-Glo MT cell viability assay (Promega, Southampton, UK) was used to measure the effect of ACR on primary Müller cell survival in the absence and presence of 2-HDP. Full details of the assay are provided in the ESM Methods.

### Confocal immunolabelling

Immunofluorescent staining of retinal cryosections was performed as previously described [5, 8]. Retinal sections were stained for FDP-lysine, GFAP, haem oxygenase-1 (HO-1), inwardly rectifying K^+^ channel 4.1 (Kir4.1), receptor for advanced glycation end-products (RAGE), S100 calcium-binding protein B (S100B) and ionised calcium-binding adapter molecule 1 (IBA1). The primary and secondary antibodies used are listed in ESM Table 1. Full details of the confocal immunolabelling methods are provided in the ESM Methods.

### Liquid chromatography high-resolution mass spectrometry

Diabetic rats administered clean drinking water (control) or water containing 2-HDP (100 mg/l) were killed after 1 week, their retinas dissected, flash frozen and extracted with methanol. Extracted samples were dried, reconstituted in water and analysed with a Waters ACQUITY I-Class ultra-high performance liquid chromatography (UHPLC) system (Milford, MA, USA) coupled to a Waters Xevo G2-XS QTOF mass spectrometer (Manchester, UK) in the mass-to-charge ratio (m/z) range 50–1200. Full details of the liquid chromatography high-resolution mass spectrometry **(**LC-HRMS) method are provided in the ESM Methods.

### Quantitative RT-PCR

Real-time quantitative reverse transcriptase PCR (qRT-PCR) was performed on mRNA from individual rat retinas. The genes analysed were: interleukin 1β *(Il1b),* chemokine (C-C motif) ligand 2 (*Ccl2*) and intercellular adhesion molecule 1 (*Icam1*). Primer sequences are presented in ESM Table 2. Full details of the qRT-PCR methods are provided in the ESM Methods.

### Electroretinography

To avoid possible attenuation of the stimulus due to cataract formation, scotopic electroretinograms (ERGs) were recorded 7 weeks after diabetes induction using an EPIC-4000 Visual Electrodiagnostic Testing System (LKC Technologies, Gaithersburg, MD, USA). Rats were dark adapted overnight, anaesthetised with ketamine/xylazine (150 mg/20 mg per kg body weight) and the pupils dilated using 1% atropine and 2.5% phenylephrine eye drops (Chauvin Pharmaceuticals, Kingston upon Thames, UK). Electrical responses were recorded with a DTL Plus corneal electrode (Diagnosys, Littleton, MA, USA), with a reference electrode placed subcutaneously in the frontal portion of the scalp and a ground electrode in the tail. White light flashes from a handheld Mini Ganzfeld system (CMGS–1; LKC Technologies) were used as the light stimulus. The photostimulator unit was calibrated to deliver 2.5 cd s/m^2^ at 0 dB flash intensity and scotopic measurements were recorded with flash intensities increasing from −25 dB to +10 dB. The ERG waveforms were recorded with a bandwidth of 0.3 to 500 Hz and sampled at 2 kHz by a digital acquisition system. Five responses were averaged at each light intensity to obtain a single ERG recording. The amplitudes for the a- and b-waves were measured. The oscillatory potentials were isolated at +10 dB stimulus intensity and the summed oscillatory potential time to peak calculated.

### Statistical analysis

Data are presented as mean ± SEM. Statistical analyses were performed using Prism V5.03 (Graphpad, San Diego, CA, USA). All data were checked for normality using Kolmogorov–Smirnov tests and non-parametric tests were applied when they did not follow a normal distribution. Two-tailed Student’s *t* tests were used to compare differences between two samples. Differences between all other datasets were analysed by one-way ANOVA with Newman–Keuls post-hoc test, Kruskal–Wallis with Dunn’s post-hoc test or multi-way ANOVA (ACR-scavenging ELISA and ERG data) with Bonferroni’s post-hoc test, as appropriate.

## Results

### 2-HDP is a potent ACR scavenger that prevents Müller cell FDP-lysine accumulation during diabetes

**Fig. 1:**
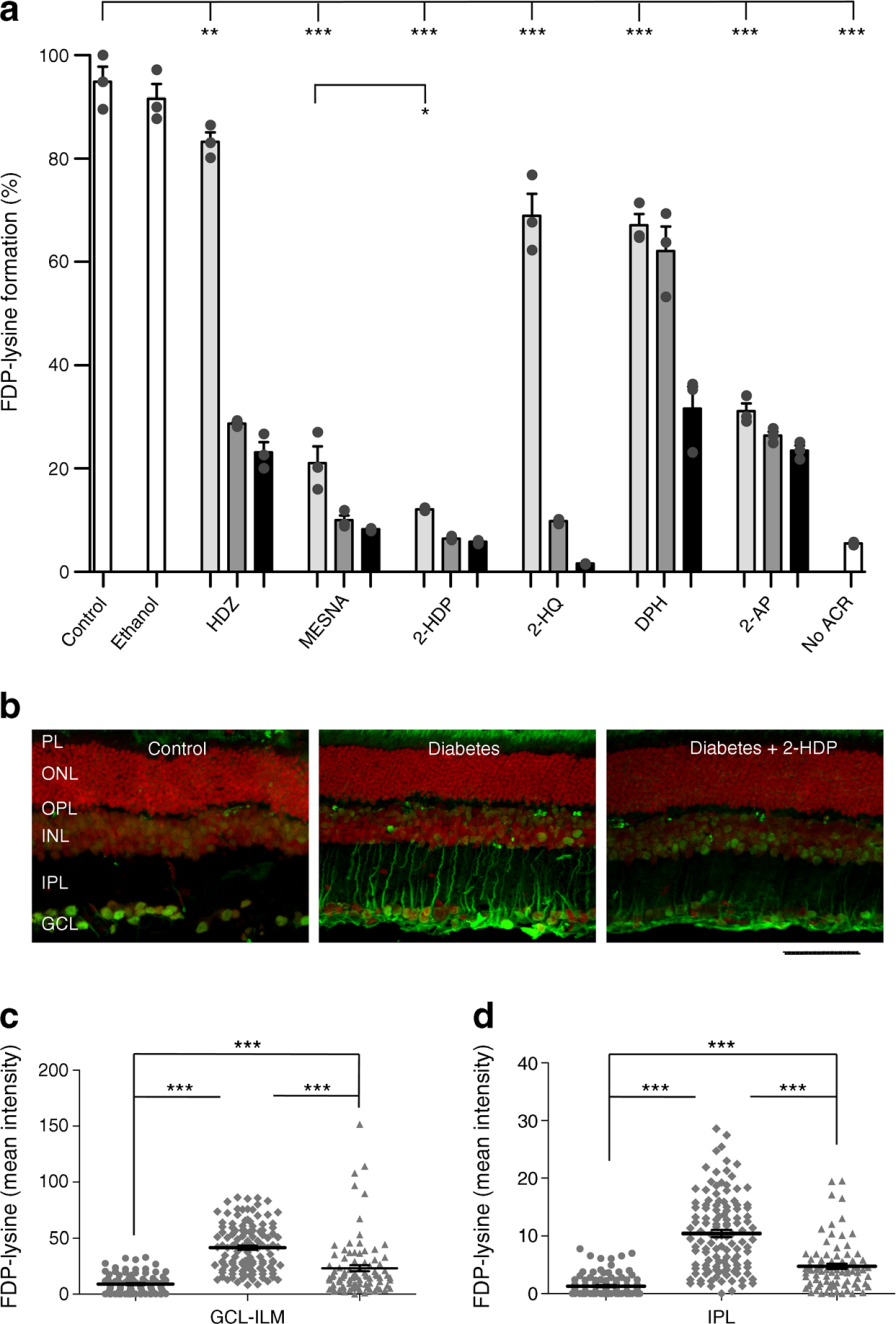
(**a**) FDP-lysine detected using ELISA following conjugation with ACR in the presence of three concentrations of different scavenging molecules (light grey bars, 1.25 mmol/l; medium grey bars, 2.5 mmol/l; black bars, 5 mmol/l), expressed as a percentage of control (no scavenger present; PBS+ACR; data were normalised to the first control replicate). The scavenging molecules tested were: HDZ; MESNA; 2-HDP; 2-HQ; DPH; and 2-AP. Experiments were carried out in triplicate; ethanol (50% ethanol + ACR) and samples with no added ACR (No ACR; PBS only) were included as additional controls. (**b**) FDP-lysine immunolabelling (green) on retinal cross sections of control, diabetic and 2-HDP-treated diabetic animals; scale bar, 50 μm. Cell nuclei are counterstained with propidium iodide (red). (**c**, **d**). Column scatter graphs showing mean pixel intensity from regions of interest randomly selected within the ganglion cell layer-inner limiting membrane (**c**) and inner plexiform layer (**d**). Circles, control; diamonds, diabetes; triangles, diabetes +2-HDP; *n*=6 retinas from six animals in each group. *p<0.05, **p<0.01 and ***p<0.001 for the indicated comparisons. GCL, ganglion cell layer; ILM, inner limiting membrane; INL, inner nuclear layer; IPL, inner plexiform layer; ONL, outer nuclear layer; OPL, outer plexiform layer; PL photoreceptor layer

Until now, few drugs capable of preventing the damaging effects of cellular ACR and FDP-lysine accumulation have been identified. Here, we sought to explore the potential of agents capable of directly scavenging ACR as therapeutic agents for diabetic retinopathy. In vitro ACR scavenging by HDZ and MESNA was first compared with that of a range of chemicals selected as having appropriate structures and a partition coefficient (log *P* −0.4 to +5.6), suggesting they could cross physiological barriers [18]. The drugs tested were 2-HDP, 2-HQ, DPH and 2-AP. These were mixed with 5 mmol/l ACR at three different concentrations (1.25 mmol/l, 2.5 mmol/l and 5 mmol/l) and formation of FDP-lysine on HSA evaluated after 1 h incubation using an ELISA-based system. All the agents tested produced reductions in FDP-lysine at each concentration tested (Fig. 1a). The most effective scavenger of ACR at the lowest concentration used (1.25 mmol/l) was 2-HDP (*p* < 0.05 vs MESNA at this concentration), with levels of FDP-lysine similar to those seen in the control group with no added ACR (Fig. 1a; *p* > 0.05 vs no ACR). Ethanol (50%) was included as a vehicle control for DPH and 2-HQ and had no effect on FDP-lysine formation.

To further confirm that 2-HDP acts as a potent ACR scavenger, we examined the ability of this compound to confer protection against ACR-induced Müller cell death in vitro. Exposure of primary mouse Müller cells for 24 h to increasing concentrations of ACR resulted in a concentration-dependent decrease in cell survival with an IC_50_ of 20 μmol/l (ESM Fig. 1a). We found that 2-HDP protected against ACR (20 μmol/l)-induced cell death in a concentration-dependent manner (ESM Fig. 1b). Significant protection was observed with 2-HDP concentrations ≥40 μmol/l (ESM Fig. 1b).

Having identified 2-HDP as a potent ACR-scavenging agent, we proceeded to investigate whether this drug is capable of preventing Müller cell FDP-lysine accumulation during experimental diabetes in rats. Accumulation of FDP-lysine in diabetic Müller glia was initially confirmed by co-labelling retinal sections from control and diabetic rats for FDP-lysine and GFAP as described previously [9] (ESM Fig. 2). When administered to diabetic rats in their drinking water (100 mg/l), 2-HDP appeared to be well tolerated and had no effect on glycaemic control, water consumption or body weight when compared with non-treated diabetic animals (*p* > 0.05; Table 1). Based on recorded fluid consumption, the average daily dose of 2-HDP was 21 mg. This substantially reduced FDP-lysine accumulation in retinal Müller cells after 3 months’ duration of diabetes (Fig. 1b–d). In animals treated with 2-HDP, FDP-lysine antibody staining in the region of the ganglion cell layer and inner limiting membrane was significantly less intense than in the diabetic animals (*p <* 0.001), but it was still higher than in the control group. This pattern was repeated in the inner plexiform layer, where Müller cell processes were more intensely stained with the FDP-lysine antibody in the diabetic animals compared with control animals. This staining was reduced in the 2-HDP-treated diabetic animals (*p <* 0.001).

**Table 1:** Diabetes-related characteristics of the three experimental groups studied

Variable	Control (n=9)	Diabetic (n=7)	Diabetic +2-HDP (n=7)
Daily fluid consumption (ml)	55.5 ± 2.6	228.4 ± 4.6***	209.4 ± 5.1***
Start weight (g)	238.9 ± 7.6	227.5 ± 4.6	206.6 ± 14.6
End weight (g)	527.7 ± 14.7	219.1 ± 8.6***	218.6 ± 13.4***
HbA_1c_ (mmol/mol)	44 ± 13	121 ± 8***	123 ± 15***
HbA_1c_ (%)	6.2 ± 1.2	13.2 ± 0.7***	13.4 ± 1.36***

Data are mean ± SEM

Fluid consumption was measured daily over 1 month for each animal

***p<0.001 vs control animals

### 2-HDP adduct is detectable in the retina on 2-HDP supplementation

**Fig. 2:**
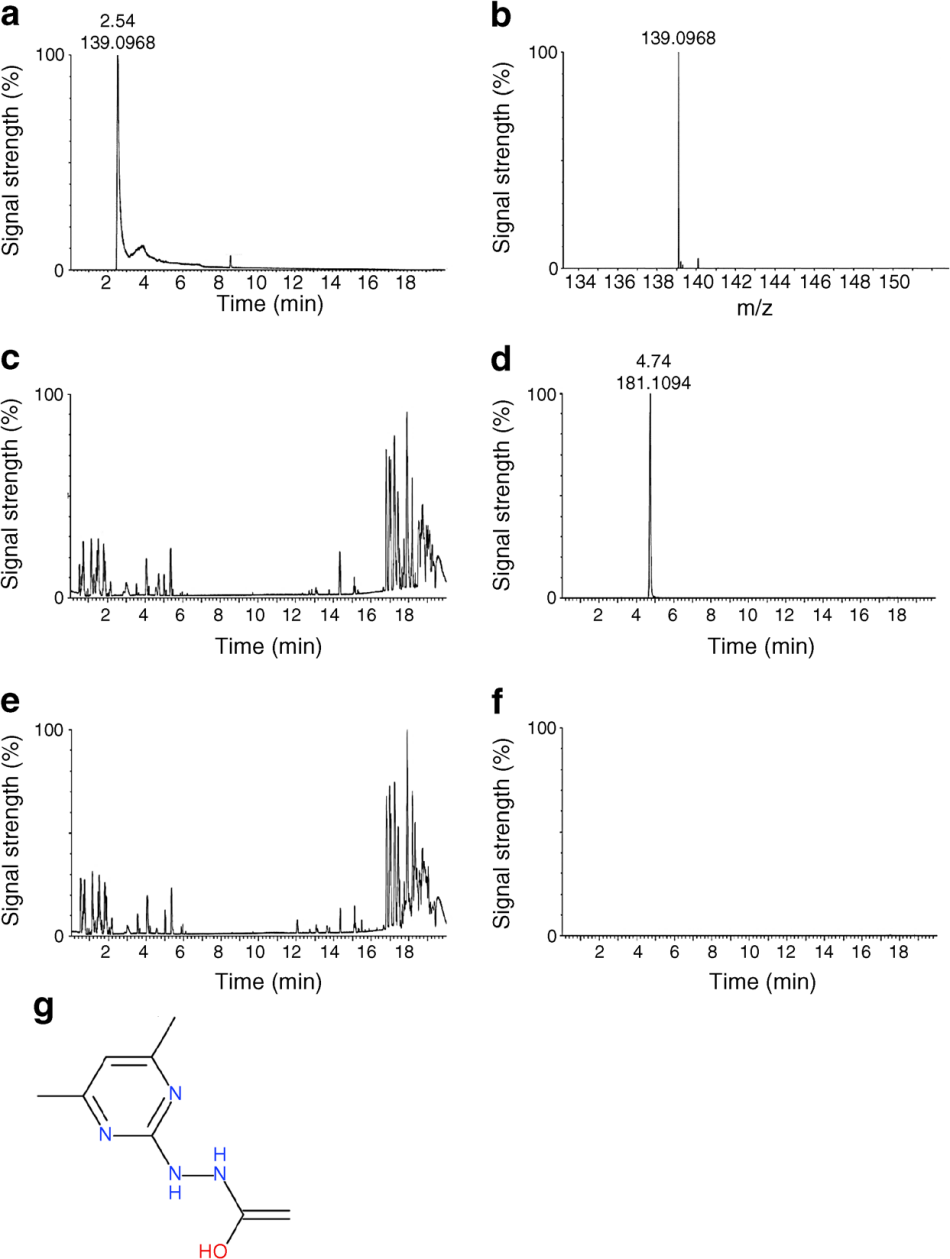
(**a**) Chromatogram of 2-HDP standard. (**b**) Mass spectrogram of 2-HDP standard. (**c**) Chromatogram of a retinal sample from a diabetic rat given 2-HDP in its drinking water (100 mg/l) for 1 week. (**d**) Extracted chromatogram from (**c**) for 139.10 m/z showing detection of a 2-HDP-related compound eluting at 4.74 min retention time. (**e**) Chromatogram of a retinal sample from a diabetic rat not administered 2-HDP. (**f**) Extracted chromatogram from (**e**) for 139.10 m/z showing the absence of a 2-HDP-related peak. (**g**) Proposed structure of the molecule found at 4.74 min retention, 181.11 m/z and deduced composition of: C_8_H_13_N_4_O

We used LC-HRMS to detect the presence of 2-HDP and related compounds in the retinas of diabetic rats given 2-HDP in their drinking water. The chromatogram (Fig. 2a) and mass spectrum (Fig. 2b) show the 2-HDP standard to peak at 2.54 min with an m/z of 139.10. In retinal samples from animals treated with 2-HDP, we detected the presence of a related compound at 4.74 min retention (Fig. 2c) and 181.11 m/z (Fig. 2d). This compound was absent from retinas of diabetic animals not treated with 2-HDP (Fig. 2e,f). Elemental composition analysis of this compound provided the formula C_8_H_13_N_4_O, based on which a plausible structure is shown in Fig. 2g.

### 2-HDP protects against Müller cell gliosis, oxidative stress and K^+^ channel dysregulation

**Fig. 3:**
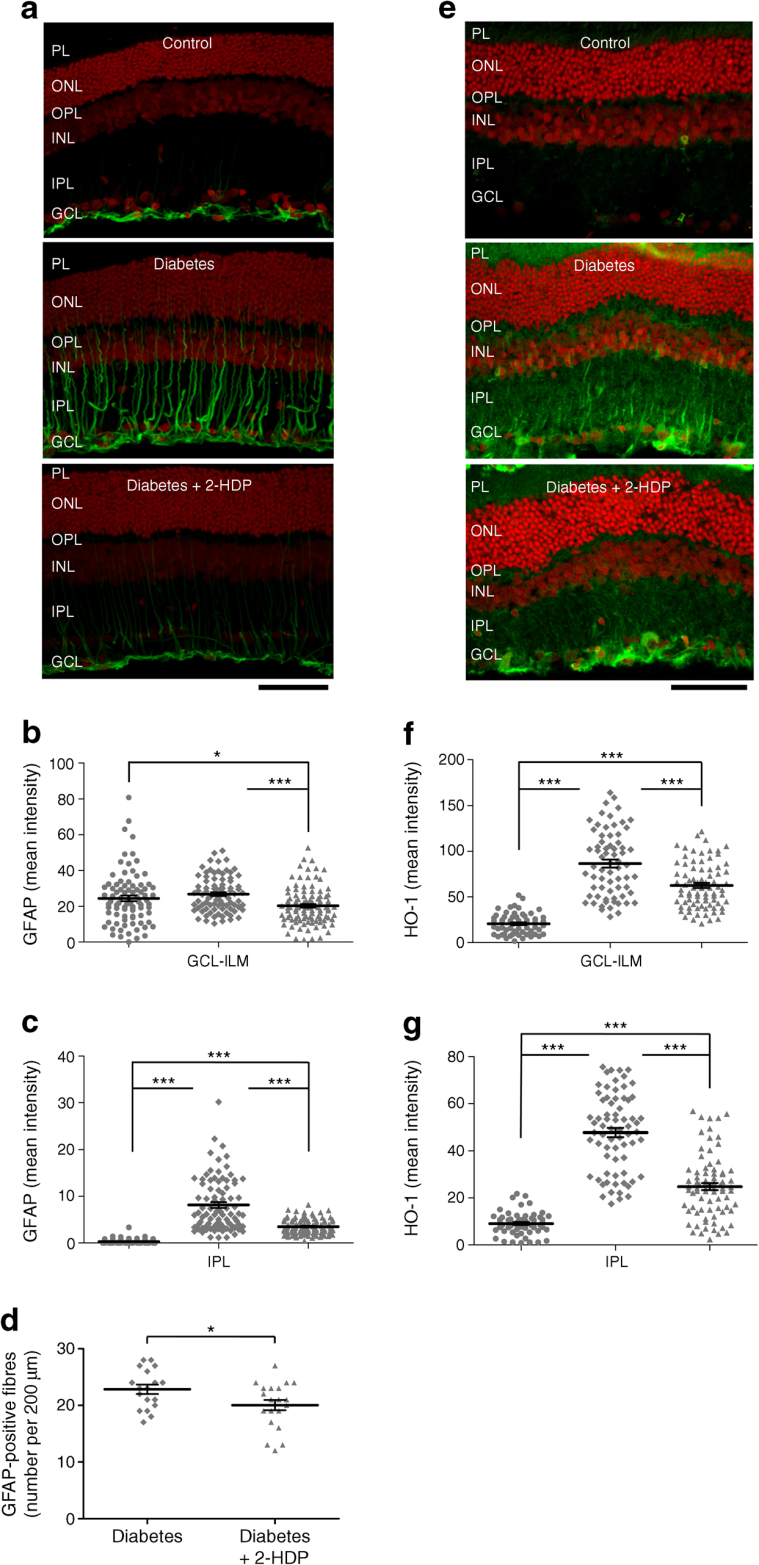
(**a**) GFAP immunolabelling (green) on retinal cross sections of control, diabetic and 2-HDP-treated diabetic animals; scale bar, 50 μm. Cell nuclei are counterstained with propidium iodide (red). (**b**, **c)** Column scatter graphs for the mean GFAP-staining intensity in regions of interest randomly selected within the ganglion cell layer-inner limiting membrane (**b**) and inner plexiform layer (**c**). Circles, control; diamonds, diabetes; triangles, diabetes + 2-HDP. (**d**) Column scatter graph showing the mean density of GFAP-labelled Müller cell fibres. *n*=6 retinas from six animals in each group. (**e**) Immunolabelling for HO-1 (green) in each of the three groups; scale bar, 50 μm. Cell nuclei are counterstained with propidium iodide (red). (**f**, **g**). Column scatter graphs for the mean HO-1-staining intensity in regions of interest randomly selected within the ganglion cell layer-inner limiting membrane (**f**) and inner plexiform layer (**g**). Circles, control; diamonds, diabetes; triangles, diabetes + 2-HDP; *n*=6 retinas from six animals in each group; a minimum of two and maximum of three retinal sections were analysed per animal. *p<0.05 and ***p<0.001 for the indicated comparisons. GCL, ganglion cell layer; ILM, inner limiting membrane; INL, inner nuclear layer; IPL, inner plexiform layer; ONL, outer nuclear layer; OPL, outer plexiform layer; PL photoreceptor layer

To explore whether limiting ACR/FDP-lysine accumulation using 2-HDP is capable of preventing Müller cell dysfunction during diabetes, we began by evaluating the effects of this drug on the Müller cell gliotic response. Diabetes had no effect on expression of the gliotic marker GFAP in the ganglion cell layer and adjacent inner limiting membrane (Fig. 3a,b), but clearly upregulated expression in the retinal Müller cell fibres within the inner plexiform layer (Fig. 3a,c). This response was attenuated by 2-HDP, as indicated by a decrease in the intensity of GFAP staining (*p <* 0.001) and a small reduction in the number of GFAP-positive fibres in the inner plexiform layer (Fig. 3a,c,d; *p* < 0.05).

Oxidative stress in the Müller cells also appeared to be reduced effectively by 2-HDP. The oxidative stress marker, HO-1 [19], was elevated in the inner retinal layers of diabetic animals in a localisation pattern consistent with Müller glia (Fig. 3e,g), as we have previously described [8]. Diabetes-induced upregulation of HO-1 was significantly reduced by 2-HDP in both the ganglion cell and inner plexiform layers (*p <* 0.001 for both comparisons), although staining remained above control levels (Fig. 3e–g).

**Fig. 4:**
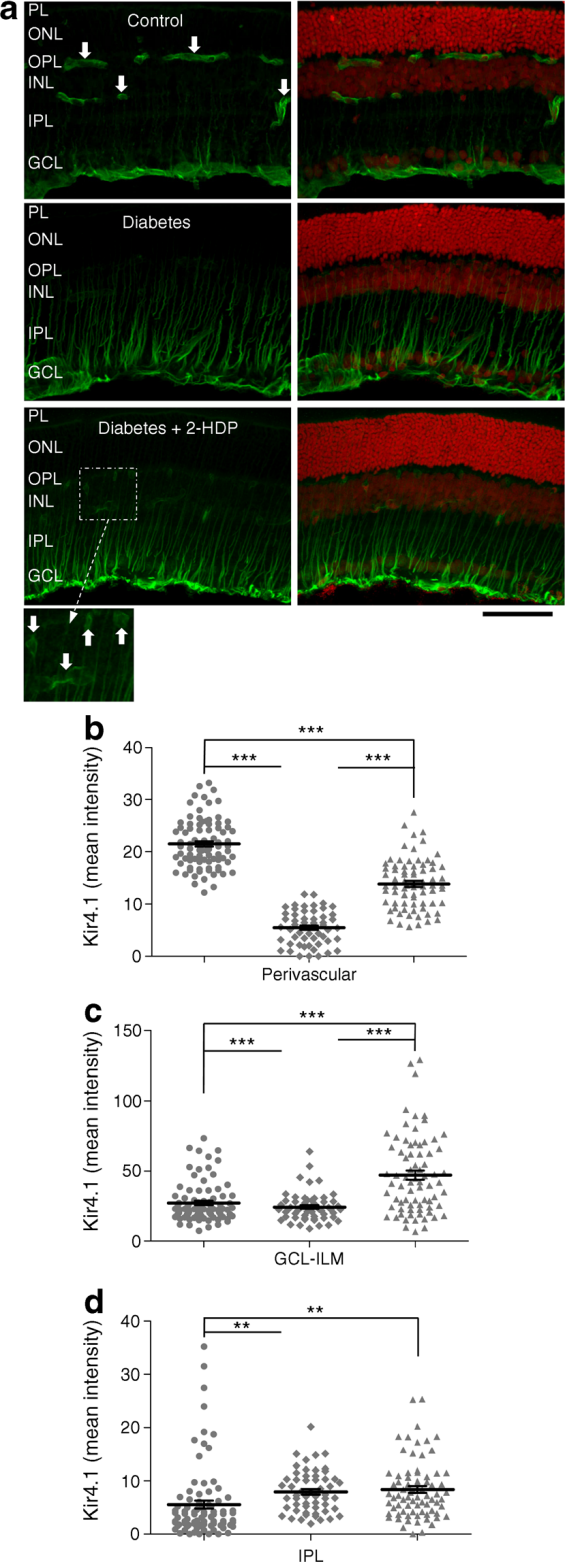
(**a**) Immunolabelling for Kir 4.1 (green) shown both with and without the red channel (propidium iodide). Perivascular distribution of Kir4.1 in the outer and inner plexiform layers is indicated (white arrows). This pattern was not seen in sections from diabetic animals but was partly restored in diabetic animals treated with 2-HDP (magnified inset); scale bar, 50 μm. (**b–d**) Column scatter graphs show mean stain intensity for perivascular (**b**), ganglion cell layer-inner limiting membrane (**c**) and inner plexiform layer (**d**) regions of interest. Circles, control; diamonds, diabetes; triangles, diabetes + 2-HDP; *n*=6 retinas from six animals in each group; a minimum of two and maximum of three retinal sections were analysed per animal; **p<0.01 and ***p<0.001 for the indicated comparisons. GCL, ganglion cell layer; ILM, inner limiting membrane; INL, inner nuclear layer; IPL, inner plexiform layer; ONL, outer nuclear layer; OPL, outer plexiform layer; PL photoreceptor layer

It is recognised that diabetes disturbs the localisation of Kir4.1 channels in Müller cells [8, 20]. In the healthy retina, Kir4.1 is expressed at the highest levels in Müller cell end-feet at the inner limiting membrane and in perivascular regions of the intermediate and deep capillary plexuses. In diabetes, Kir4.1 expression is lost from the perivascular and inner limiting membrane regions. This is thought to disrupt Müller-cell-mediated K^+^ clearance in the retina, leading to extracellular K^+^ accumulation, excitotoxicity and impaired fluid transport [20]. We observed a diabetes-induced reduction in Kir4.1 immunoreactivity in the perivascular areas and in the innermost region of the ganglion cell layer corresponding to the inner limiting membrane, as described in these previous reports [8, 20] (Fig. 4a–c). The effects of diabetes on the distribution of Kir4.1 channels were partially reversed in the perivascular areas and completely reversed in the ganglion cell layer following treatment with 2-HDP (*p <* 0.001 in both cases), where a significant increase in staining was seen relative to non-diabetic control animals (Fig. 4a–c). Kir4.1 immunoreactivity in the Müller cell fibres within the inner plexiform layer was increased in sections from diabetic animals and this was unaffected by 2-HDP (Fig. 4a,d).

### 2-HDP regulates Müller-cell-mediated pro-inflammatory responses in the diabetic retina

**Fig. 5:**
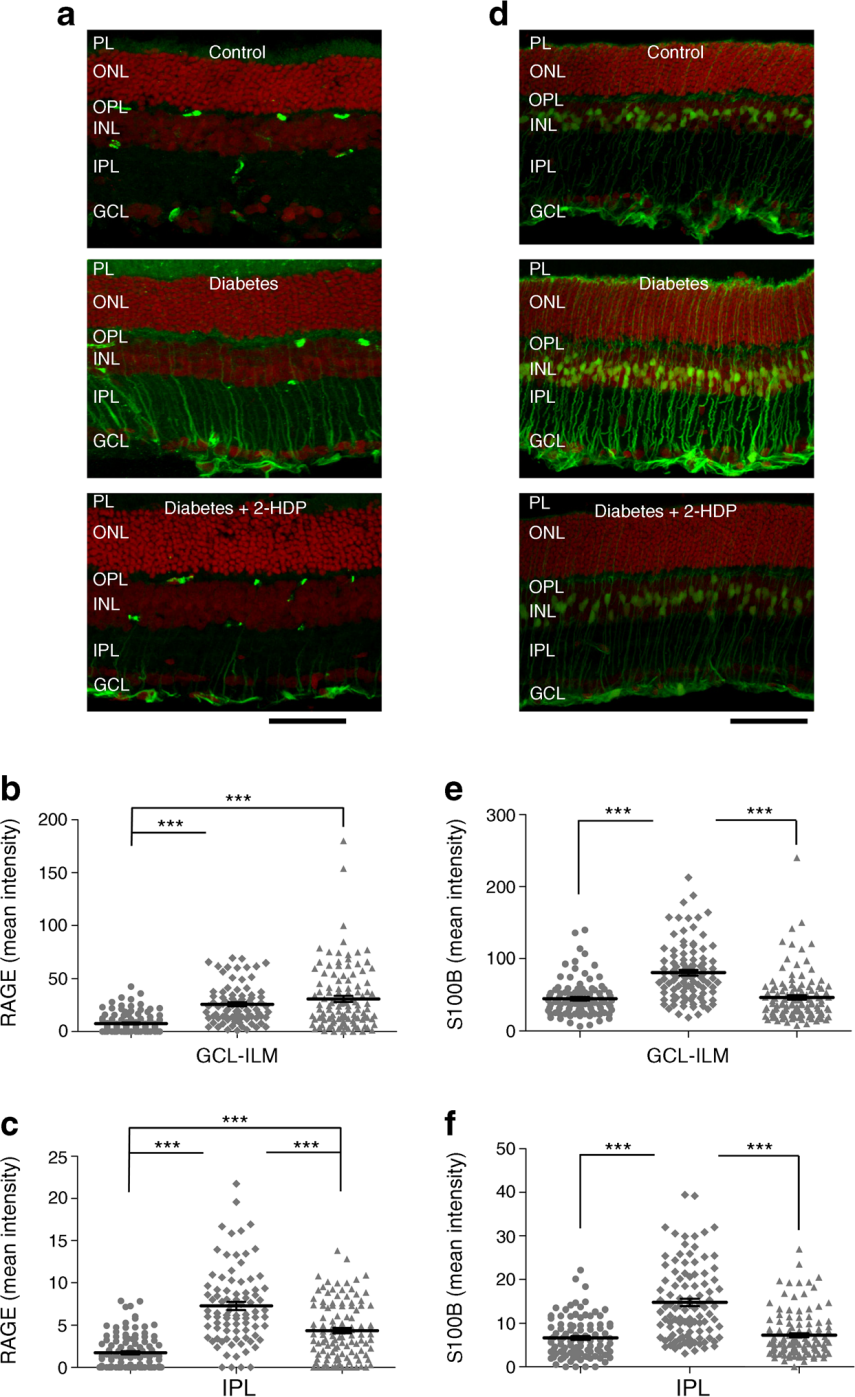
(**a**) Immunolabelling (green) for RAGE in retinal sections from control and diabetic animals, and from diabetic animals treated with 2-HDP; scale bar, 50 μm. (**b**, **c**) Column scatter graphs showing the mean pixel fluorescence intensity for RAGE in ganglion cell layer-inner limiting membrane (**b**) and inner plexiform layer (**c**) regions of interest. Circles, control; diamonds, diabetes; triangles, diabetes + 2-HDP; *n*=6 retinas from six animals in each experimental group. (**d**) S100B immunolabelling in each of the three groups. Scale bar = 50 μm. (**e**, **f**). Column scatter graphs representing mean S100B-staining intensity in regions of interest randomly selected within the ganglion cell layer-inner limiting membrane (**e**) and inner plexiform layer (**f**). Circles, control; diamonds, diabetes; triangles, diabetes + 2-HDP; *n*=6 retinas from six animals in each group. ***p<0.001 for the indicated comparisons. GCL, ganglion cell layer; ILM, inner limiting membrane; INL, inner nuclear layer; IPL, inner plexiform layer; ONL, outer nuclear layer; OPL, outer plexiform layer; PL photoreceptor layer

It has previously been demonstrated that the pattern recognition receptor, RAGE, is upregulated in retinal Müller cells after 3 months of diabetes, together with one of its major ligands, S100B [5]. Upregulation of RAGE and S100B has previously been linked to the activation of microglia and the induction of Müller cell inflammatory cytokine expression in diabetes [5, 21]. In the present work, we observed strong upregulation of RAGE and S100B in retinal Müller cells of diabetic rats (Fig. 5a–f). With the exception of RAGE expression at the ganglion cell layer and inner limiting membrane, these effects were reduced or abolished by treatment of the animals with 2-HDP (Fig. 5a–f).

**Fig. 6:**
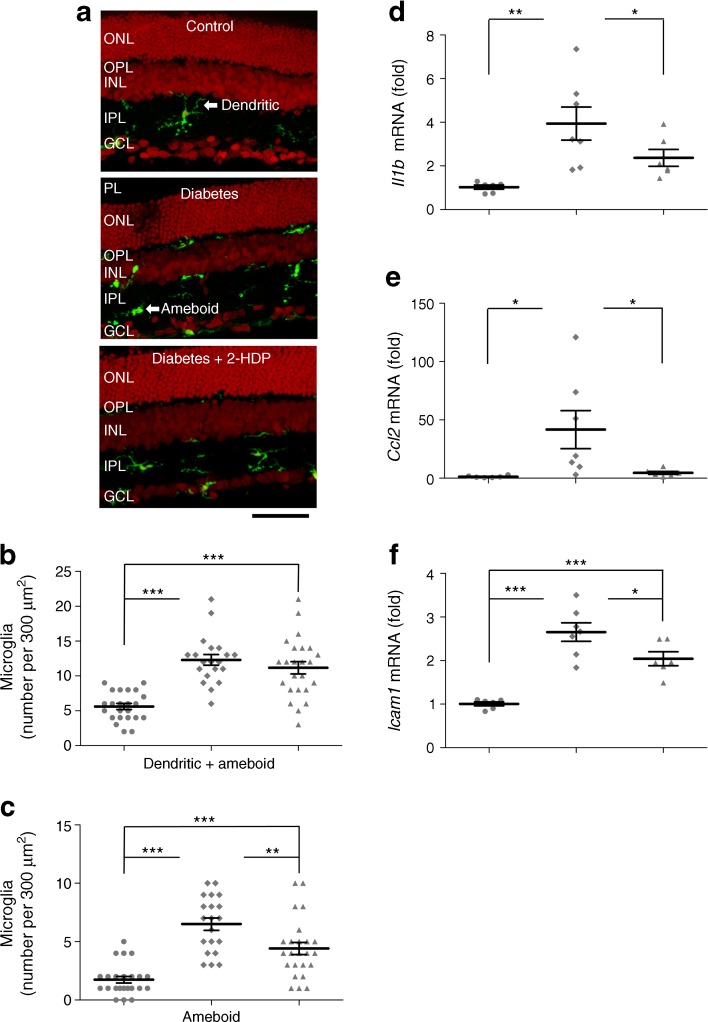
(**a**) IBA1-immunolabelled microglia (green) in retinal sections. Microglia demonstrated both dendritic and amoeboid morphologies; scale bar, 50 μm. (**b**, **c**) Scatter plots showing total microglial density (**b**) and the density of amoeboid microglia (**c**) in sections from control animals (circles), animals with diabetes alone (diamonds) and diabetic animals (triangles) treated with 2-HDP; *n*=6 retinas from six animals in each experimental group. (**d**–**f**) Column scatter graphs summarising retinal mRNA expression for the inflammatory signalling molecules *Il1b* (**d**), *Ccl2* (**e**) and *Icam1* (**f**), expressed as a ratio of expression in control tissues. Circles, control; diamonds, diabetes; triangles, diabetes + 2-HDP; *n*=6 control and diabetes + 2-HDP retinas from six animals; *n*=7 diabetic retinas from seven animals. *p<0.05, **p<0.01 and ***p<0.001 for the indicated comparisons. GCL, ganglion cell layer; INL, inner nuclear layer; IPL, inner plexiform layer; ONL, outer nuclear layer; OPL, outer plexiform layer; PL, photoreceptor layer

Retinal sections from diabetic animals immunolabelled with the microglial marker IBA1 appeared to have many more microglia than those of control animals (Fig. 6a). The fraction of microglia displaying an ‘ameboid’, or activated phenotype [22], also increased from 0.355 ± 0.062 in sections from control animals to 0.534 ± 0.034 in sections from diabetic rats (*p* < 0.01). Diabetic animals treated with 2-HDP still exhibited an increase in the number of microglia present, but the proportion of activated microglia was decreased to 0.348 ± 0.030 when compared with untreated diabetic rats (*p* < 0.05; Fig. 6b,c).

In line with our observation of increased numbers of activated microglia, the inflammatory markers *Ccl2*, *Il1b* and *Icam1* were transcriptionally upregulated in the retinas of diabetic animals (Fig. 6d–f). Treatment of diabetic rats with 2-HDP caused a significant reduction in the mRNA levels of *Ccl2*, *Il1b* and *Icam1* (Fig. 6d–f).

### 2-HDP improves retinal neurophysiological function in diabetes

**Fig. 7:**
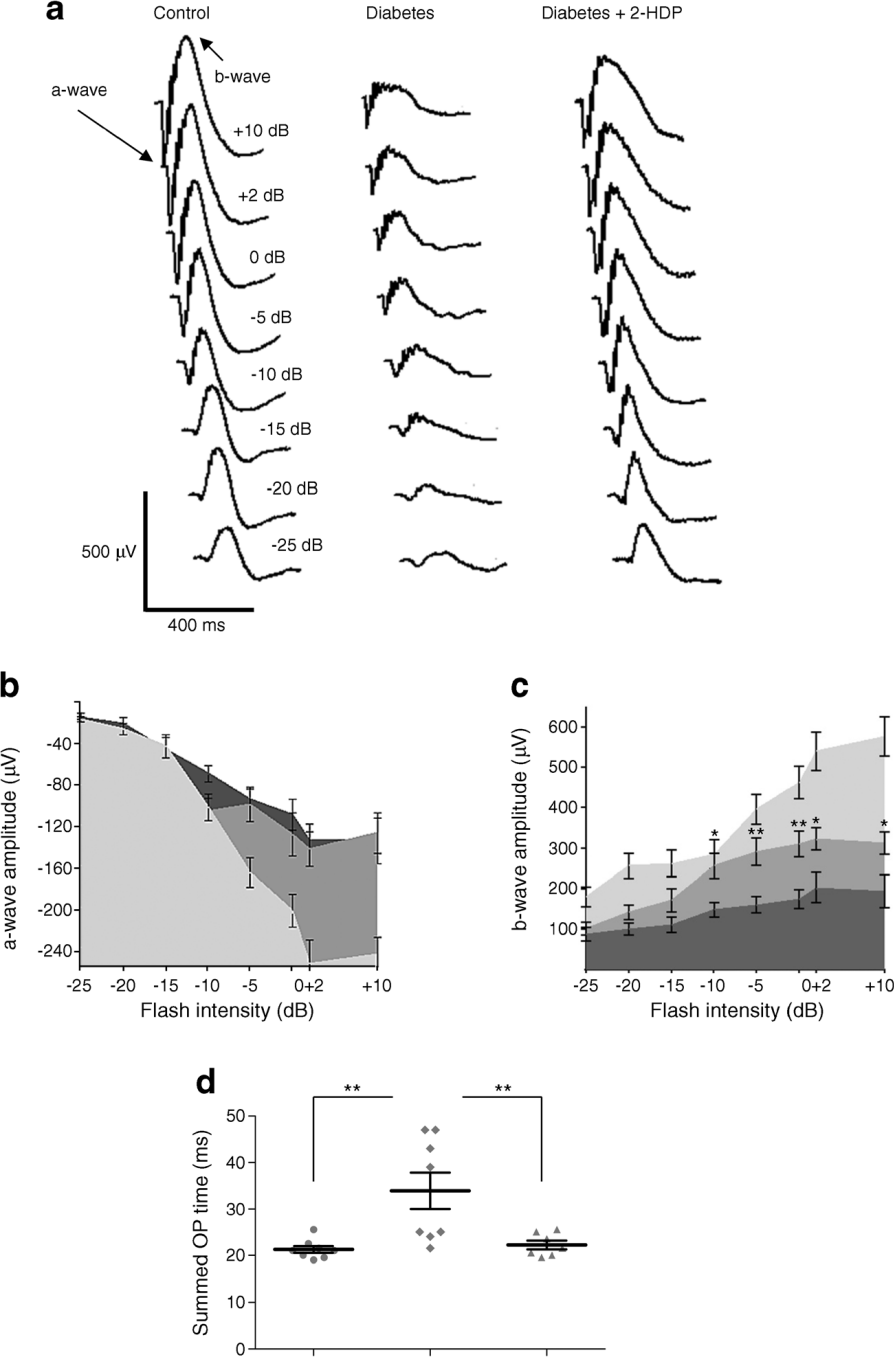
(**a**) Representative scotopic ERG waveforms at a range of stimulus intensities from −25 Db to +10 Db for a control, diabetic and 2-HDP-treated diabetic animal. The a- and b-waves are labelled for the control response elicited at +10 Db. (**b**, **c**) Summary data for a-wave (**b**) and b-wave (**c**) amplitudes for control (light grey), diabetic (dark grey) and diabetic animals treated with 2-HDP (medium grey). For clarity, only significant differences between diabetic and 2-HDP-treated diabetic animals are indicated (*p<0.05 and **p<0.01 vs diabetes alone). (**d**) Scatter plot showing data for summed oscillatory potential times (**p<0.01 for the indicated comparisons). *n*=8 animals in the control and diabetic groups and *n*=7 animals in the diabetic + 2-HDP group. OP, oscillatory potential

Müller cell dysfunction is believed to contribute to alterations in the ERG in diabetic animals [23]. Previous studies have shown that the a- and b-waves are reduced in amplitude and the kinetics of the oscillatory potentials are slowed in rat models of diabetes [24, 25]. These findings were confirmed in the current study. Both the a- and b-waves were significantly depressed at 7 weeks in diabetic rats compared with control rats across a wide range of light stimulus intensities (*p* < 0.001 for diabetes vs control at intensities ≥−5 Db for a-wave amplitude and at all intensities for b-wave amplitude; Fig. 7a–c). Diabetic animals treated with 2-HDP showed a significant increase in b-wave amplitude at intensities ≥−10 Db (Fig. 7a,c; *p* < 0.05–0.01). Treatment with 2-HDP had no effect on the a-wave amplitude. Diabetes slowed the oscillatory potentials, increasing the summed oscillatory potential time to peak. Treatment with 2-HDP prevented this (Fig. 7d; *p* < 0.01).

## Discussion

Oxidative stress results in the formation of lipid aldehydes, which covalently bind to nucleophilic amino-acid residues to form adducts known as advanced lipoxidation end-products (ALE) [26]. This process has been implicated in the pathogenesis of a range of ocular diseases, including diabetic retinopathy (see review by McDowell et al [10]). We have previously shown that the ACR-derived FDP-lysine adduct accumulates on Müller cell proteins during diabetes [8, 9]. Our most recent studies suggest that this may reflect downregulation of aldehyde dehydrogenase 1A1 (ALDH1A1) in the diabetic retina, leading to reduced aldehyde detoxification [27]. FDP-lysine accumulation within Müller cells appears to contribute to the dysfunction of these cells during the early stages of experimental diabetic retinopathy [8, 9]. This suggests that reducing ACR levels may mitigate against the development of retinopathy in people with diabetes [10].

In the current study, we have explored the therapeutic potential of this approach using the hydrazino compound 2-HDP. In vitro, 2-HDP was a more potent ACR-scavenging agent than both the anti-hypertensive parent compound, HDZ and MESNA, a thiol-based ACR scavenger used acutely to reduce the side effects of alkylating chemotherapeutic drugs [11]. In our hands, it blocked ACR-induced Müller cell death in culture. In vivo, 2-HDP was capable of preventing the accumulation of FDP-lysine in retinal Müller glia of diabetic rats. A stable 2-HDP adduct was found in the retina of diabetic rats treated with this compound. While the exact route of formation of this 2-HDP adduct remains to be elucidated, it would be consistent with the generation of a 2-HDP–ACR adduct subjected to oxidative decarboxylation. Supplementation with 2-HDP was associated with reduced Müller cell gliosis, oxidative stress and pro-inflammatory signalling as well as improvements in Kir4.1 localisation and retinal neurophysiological function.

These findings provide preclinical evidence that 2-HDP in particular and ACR scavengers in general may offer novel therapeutic options for diabetic retinopathy. They also support a role for both ALEs and inflammation in the disease process. In recent years, the importance of retinal inflammation in the pathogenesis of diabetic retinopathy has become increasingly clear (see recent review by Roy et al [28]). Glial-derived inflammatory cytokines are upregulated in people with diabetes [29] and Müller glia are deemed to be one of the key cell types responsible for the upregulation of pro-inflammatory cytokines in the diabetic retina [5]. Previous reports have shown that hyperglycaemia is associated with oxidative stress and pro-inflammatory signalling in Müller cells [5, 8, 9]. Inflammatory signalling in the diabetic retina was suppressed by 2-HDP in the current study, supporting the hypothesis that accumulation of ACR/FDP-lysine adducts in Müller glia plays a significant role in the development of retinal inflammation during diabetes. Taken together with our data on retinal aldehyde detoxification in diabetes [27], these findings suggest that hyperglycaemia downregulates retinal ALDH1A1 expression, leading to ACR/FDP-lysine accumulation within Müller cells and resulting in oxidative stress and inflammation. ACR/FDP-lysine accumulation is known to induce oxidative stress through the enhancement of lipid peroxidation reactions and the depletion of endogenous antioxidants such as glutathione [30]. The observation that scavenging of ACR with 2-HDP was associated with reduced expression of HO-1 is consistent with this.

The changes seen in the ERG at 7 weeks’ diabetes were similar to those reported in other animal studies, with reductions in the a- and b-waves and slowed oscillatory potential kinetics [24, 25]. Inhibition of the oscillatory potentials is reported early in human diabetes [31, 32], and reductions in b-wave amplitude are also seen [33], though less consistently (for reviews, see Pescosolido et al [34] and Tzeko and Arden [35]). Treatment of animals with 2-HDP significantly increased b-wave amplitudes compared with their non-treated counterparts and prevented oscillatory potential slowing. Although it has been postulated that Müller cells may contribute directly to the ERG b-wave [36], it is now generally accepted that light-induced activity of ON-bipolar cells is the key b-wave determinant [37, 38], while oscillatory potentials probably reflect activity of amacrine and ganglion cells [39]. This indicates that 2-HDP treatment improves inner retinal neurophysiology, consistent with inhibition of ACR/FDP-lysine accumulation in Müller cells and resultant pro-inflammatory signalling. Of course, direct effects of 2-HDP on other retinal cell types during diabetes cannot be discounted. The fact that 2-HDP did not alter the effects of diabetes on the a-wave amplitude indicates that the partial normalisation of the b-wave and oscillatory potentials most likely results from improved inner retinal function and is not simply secondary to changes in photoreceptor activity [40].

Given the wide range of transcription effects induced by ACR in cell culture [41] it is not surprising that there is growing evidence of a therapeutic role for ACR scavengers in a wide range of pathologies. Hydralazine and phenelzine (a hydrazine derivative) have been shown to reduce ACR adducts, tissue damage and neuropathic pain behaviours in rat model of spinal cord injury [42, 43]. *N*-acetyl-l-cysteine (NAC), MESNA, mercaptamine and N-benzylhydroxylamine (NBHA) all reduce ACR cell toxicity in vitro and acetaminophen liver damage in vivo in mice [44, 45], although NAC had no beneficial effects on ACR-induced lung damage in rats [46]. Several carbonyl scavengers (bisulphite, d-penicillamine, hydralazine and 1-hydrazinoisoquinoline) ameliorate smoke-extract toxicity in cell culture and may have some protective effect against smoke-inhalation injury [47]. Of relevance to the present study, NBHA, by sequestering ACR, may prevent retinal pigment epithelial cell dysfunction in diabetes through a mechanism involving blockade of vascular endothelial growth factor (VEGF)/TGF-β signalling [48, 49]. The range of scavengers and possible scavenger mechanisms has been reviewed recently [10, 50].

In summary, the current study provides in vivo evidence that treatment with the ACR scavenger 2-HDP reduced FDP-lysine adduct formation in Müller cells, and was associated with reduced gliosis and a more physiological distribution of the Kir4.1 K^+^ channel in the diabetic retina. There was also reduced expression of a marker of oxidative stress and pro-inflammatory signalling molecules as well as reduced fractional activation of retinal microglia. This suppression of recognised pathogenic mechanisms was paralleled by improvements in retinal function, as indicated by the ERG. Taken together, the data suggest that the therapeutic potential of ACR scavengers in general, and 2-HDP in particular, merits further investigation to determine clinical utility in treating the retinal complications of diabetes.

## Electronic supplementary material

ESM (PDF 604 kb)

## Data Availability

The data that support the findings of this study are available from the corresponding author on reasonable request.

## Abbreviations

ACR: Acrolein
ALDH1A1: Aldehyde dehydrogenase 1A1
2-AP: 2-Aminopropanol
DPH: 1,2-Diphenylhydrazine
ERG: Electroretinogram
FDP-lysine: *N*^ε^-(3-formyl-3,4-dehydropiperidino)lysine
GFAP: Glial fibrillary acidic protein
2-HDP: 2-Hydrazino-4,6-dimethylpyrimidine
HDZ: Hydralazine
HO-1: Haem oxygenase-1
2-HQ: 2-Hydrazinoquinoline
HSA: Human serum albumin
IBA1: Ionised calcium-binding adapter molecule 1
Kir4.1: Inwardly rectifying K^+^ channel 4.1
MESNA: 2-Mercaptoethane sulfonate
m/z: Mass-to-charge ratio
NAC: *N*-acetyl-l-cysteine
qRT-PCR: Real-time quantitative reverse transcriptase PCR
RAGE: Receptor for advanced glycation end-products
STZ: Streptozotocin
S100B: S100 calcium-binding protein B
