# Supplementary material for: Müller glial dysfunction during diabetic retinopathy in rats is reduced by the acrolein-scavenging drug, 2-hydrazino-4,6-dimethylpyrimidine
Source: Diabetologia. 2018 Aug 15;61(12):2654–67. doi: 10.1007/s00125-018-4707-y (PMC6223850; doi:10.1007/s00125-018-4707-y)
Supplement: Supplementary file 1 — (PDF 604 kb) [file 125_2018_4707_MOESM1_ESM.pdf]

ESM Methods:

ELISA for drug scavenging of ACR

The ELISA was carried out by incubating ACR (Fluka, Sigma Aldrich, St. Louis, USA) and the scavenger drug under test with human serum albumin (HSA) (lyophilized; Sigma Aldrich, St. Louis, USA). Adduct formation was detected with an anti-FDP-lysine monoclonal primary antibody (Japan Institute for the Control of Aging, Japan) and horse radish peroxidase (HRP) conjugated anti-mouse secondary antibody. The fluorescent signal was measured following incubation with Quanta Blu™ fluorogenic peroxidise substrate reagent (Thermo Scientific, USA). Initial optimization experiments indicated that a reproducible but unsaturated fluorescence signal was obtained with HSA and ACR concentrations of 0.1 mg/ml and 5 mmol/l, respectively. 200 µl aliquots of 0.1 mg/ml of HSA in 50mmol/l carbonate coating buffer (36mmol/l $NaCO_3$; 14 mmol/l Na_2CO_3; pH 9.6) were placed in high binding 96 well plates (Nunc Maxisorp; Thermo Scientific, USA) and incubated at 4 °C overnight. The HSA solution was decanted and the plates washed 3 times with PBS containing 0.05% Tween-20 (PBS-T). DPH was dissolved in 50% ethanol in PBS, and 2-HQ in 0.5 ml 100% ethanol which was then made up to 10ml in PBS. Other scavenger drugs were directly dissolved in PBS. 50 µL aliquots of each scavenger were applied to each well before adding an equal volume of 5mmol/l ACR diluted in PBS, giving final drug concentrations of 1.25, 2.5 and 5mmol/l. Each plate was sealed tightly (to avoid evaporation of the volatile and toxic ACR) and incubated at 37 °C for 1hr. After this, plates were washed 3 times with PBS-T and incubated with blocking buffer (PBS with 5% bovine serum albumin (BSA), 0.05% Tween-20) for 1hr. The plates were again washed three times and the primary antibody was added (anti-FDP-lysine monoclonal antibody; 1:5000 dilution in PBS with 1% BSA and

0.05% Tween-20) and incubated for 1hr at room temperature. The plate was washed x3 again before incubation with HRP conjugated anti-mouse secondary antibody (1:10,000 dilution in 1% BSA and 0.05% Tween-20) at room temperature for 1hr. After a further three washes Quanta Blu™ Fluorogenic substrate mixture was added to the wells and allowed to develop for 30min before addition of Quanta Blu™ stop solution. The developed substrate was transferred into a clean black plate, to prevent possible detection of fluorescence from FDP-lysine or scavenger molecules, and read using a Tecan plate reader with excitation/emission settings of 325/420 nm.

Cell Survival

Primary mouse Müller cells were isolated and cultured from retinal cell suspensions as previously described [1]. Müller cells between passages 3 to 6 were grown in 96-well plates ($5x10^3$ cells per well) and cultured for 24 hours in the absence or presence of a range of ACR concentrations (1-400 μmol/l; Sigma Aldrich). Cell survival was measured using a RealTime-Glo MT Assay (Promega, Southampton, UK) kit on a FLUOstar Omega plate reader (BMG Labtech, Aylesbury, UK). The effect of 2-HDP on ACR-induced cell death was determined by pre-incubating ACR (20 μmol/l) for 1 hour with various concentrations of 2-HDP (1-400 μmol/l) prior to adding to the cells for 24 hours. For both sets of experiments, all concentrations were tested in triplicate and experiments repeated three separate times. Curve fitting and half maximal inhibitory concentrations (IC50) were determined using GraphPad Prism V5.03 (San Diego, CA). Percentage data was arcsine transformed prior to statistical analysis using one-way ANOVA with Newman-Keuls post-hoc test.

Confocal Immunolabelling

For staining of retinal cryosections, a range of primary antibodies were used in combination with appropriate fluorescently-labelled secondary antibodies (ESM

Table 1). Nuclei of retinal cells were counterstained with propidium iodide (red fluorescence; Sigma, Poole, UK). Confocal images were captured with a Nikon C1 confocal microscope (Nikon Ltd, Kingston upon Thames, UK). For quantification of fluorescence intensity within individual experiments, confocal settings were kept constant and captured images were analysed using Image J according to published protocols from our laboratory [2;3]. Fluorescence was measured for five regions of interest defined within relevant areas of each retinal section and background corrected using measurements from three non-fluorescent regions within the same section. Unless otherwise stated, a minimum of two and maximum of four retinal sections per animal were analysed and data summarised for six animals per experimental group. All secondary-only controls were negative for staining. Images were rotated and cropped for presentation purposes and the red channel (nuclear staining) adjusted to ensure that cell layers were clearly visible. No red channel data was used for quantification.

Liquid Chromatography High Resolution Mass Spectrometry (LC-HRMS)

Six diabetic rats were administered water (N=3) or 2-HDP-laced (100 mg/l) water (N=3) for one week after which they were euthanized CO_2 inhalation. Both eyes were removed and retinas dissected within 5 minutes. Retinas were then individually snap-frozen and stored at -80 °C until extraction, protected from light. Retinas were homogenized with 0.2 ml of ice cold methanol (LC–MS Chromasolv grade, Sigma-Aldrich, St Louis, MO, USA) using a hand tissue homogenizer for 30 seconds. A further 0.2 ml of ice cold methanol was then added, vortexed vigorously for 1 minute and sonicated (Camsonix C1274, Camlab, Cambridge, UK) for 10 minutes at room temperature. After centrifugation at 10,000 g for 10 minutes at 4 °C (MIKRO 200R centrifuge, Hettich UK, Salford, UK), 0.3 ml of the supernatant was transferred into a

fresh microtube and dried overnight in a miVac QUP-23050-A00 (Genevac, Ipswich, UK) centrifugal sample concentrator. The dry extracts were then reconstituted in 0.1 ml ultra-pure water and filtered through a 0.22 micrometer Costar Spin-X Centrifuge Tube Filter by centrifugation at 10,000 g for 10 minutes at 4 °C. Filtered extracts were transferred into maximum recovery LC vials (Waters, Manchester, UK) for LC-MS analysis. Analyses were carried out on a Waters Acquity UPLC I-Class system (Milford, MA, USA) coupled to a Waters Xevo G2-XS QTof mass spectrometer (Manchester, UK) with an electrospray ionization source operating in positive or negative mode with lock-spray interface for real time accurate mass correction. Instrument settings were as follow: source temperature was set at 120 °C, cone gas flow at 50 l/h, desolvation temperature at 450 °C, and desolvation gas flow at 850 l/h. The capillary voltage was set at 1.0 kV in positive mode. Source offset was 80 (arbitrary unit). Mass spectra were acquired in continuous mode using MSE function (low energy: 4 eV; high energy: ramp from 15 to 30 eV) over the range m/z 50-1200 with a scan time of 0.1 s. A lock-mass solution of Leucine Enkephalin (1 ng/µl) in methanol/water containing 0.1 % formic acid (1:1, v/v) was continuously infused into the spectrometer via the lock-spray at a flow rate of 10 µl/min.

Reverse Transcriptase Polymerase Chain Reaction

Total RNA was extracted from rat retinas using an RNeasy Mini Kit (Qiagen, Crawley, UK). The RNA concentration and purity of each sample was determined using a NanoDrop2000™ UV spectrometer (Thermo-scientific, Waltham MA, USA) and 500ng of RNA reverse transcribed into cDNA using SuperScript™ III Reverse Transcriptase Kit (Invitrogen, Life Technologies, USA). Gene specific primers were designed using NCBI Primer Blast and synthesized by Eurogentec (Southampton, UK). Primer sequences are presented in ESM Table 2. SYBR Green qRT-PCR was

performed using a ROCHE lightcycler480™ (Roche, Basel, Switzerland). Relative gene expression was calculated using the comparative Ct method ($2^{-\Delta\Delta Ct}$) with β-actin as the housekeeping gene. Minus reverse transcriptase and no template controls were negative and all PCR products were Sanger sequenced to confirm their identity.

Reference List

1. McDowell RE, McGahon MK, Augustine J, Chen M, McGeown JG, Curtis TM (2016) Diabetes Impairs the Aldehyde Detoxifying Capacity of the Retina. Invest Ophthalmol.Vis.Sci. 57: 4762-4771

2. Curtis TM, Hamilton R, Yong PH, et al (2011) Muller glial dysfunction during diabetic retinopathy in rats is linked to accumulation of advanced glycation end-products and advanced lipoxidation end-products. Diabetologia 54: 690-698

3. Yong PH, Zong H, Medina RJ, et al (2010) Evidence supporting a role for N-(3-formyl-3,4-dehydropiperidino)lysine accumulation in Muller glia dysfunction and death in diabetic retinopathy. Mol.Vis. 16: 2524-2538

ESM Tables

Target protein	Primary antibody (concentration and source)	Secondary antibody (concentration and source)
**FDP-lysine**	Mouse monoclonal antibody (mAb5F6; 1:500; JAICA)	Donkey anti-mouse AlexaFluor^{TM} 488 (1:200; Invitrogen)
**K_{ir}4.1**	Rabbit polyclonal (1:500; Alomone Labs Ltd, Israel)	Donkey anti-rabbit AlexaFluor^{TM} 488 (1:200; Invitrogen)
**GFAP**	Rabbit polyclonal (1:1000; Dako, UK)	Donkey anti-rabbit AlexaFluor^{TM} 488 (1:200; Invitrogen) (Fig 3a)<br>Donkey anti-rabbit AlexaFluor^{TM} 568 (1:200; Invitrogen) (ESM Fig 2)
**HO-1**	Rabbit polyclonal (1:50; Assay Designs, USA)	Donkey anti-rabbit AlexaFluor^{TM} 488 (1:200; Invitrogen)
**S100B**	Rabbit polyclonal (1:100; Abcam, UK)	Donkey anti-rabbit AlexaFluor^{TM} 488 (1:200; Invitrogen)
**RAGE**	Mouse monoclonal (1:200; Sigma-Aldrich, UK)	Donkey anti-mouse AlexaFluor^{TM} 488 (1:200; Invitrogen)
**IBA1**	Rabbit polyclonal (1:200; WAKO)	Donkey anti-rabbit AlexaFluor^{TM} 488 (1:200; Invitrogen)

ESM Table 1: Antibodies used in immunohistochemistry to assess retinal expression and distribution of proteins associated with oxidative stress and inflammation. Antibodies were validated by the manufacturers for immunohistochemistry analysis.

mRNA transcript	Primer sequences (Forward and Reverse)	Primer efficiency
Il1b	F: AAATGCCTCGTGCTGTCTGA<br>R: GTTTGGGATCCACACTCTCCA	1.90
Ccl2	F: CTGTAGCATCCACGTGCTGT<br>R: GGACCCATTCCTTATTGGGGT	2.09
Icam-1	F: CTTTGCCCTGGTGGTGGAAT<br>R: TGTCTTCCCCAATGTCGCTC	1.98

ESM Table 2: Primer sequences for qRT-PCR of inflammatory marker transcripts. PCR products had a melt curve with a single peak and product sizes when run out on a gel were as expected.

ESM Figures

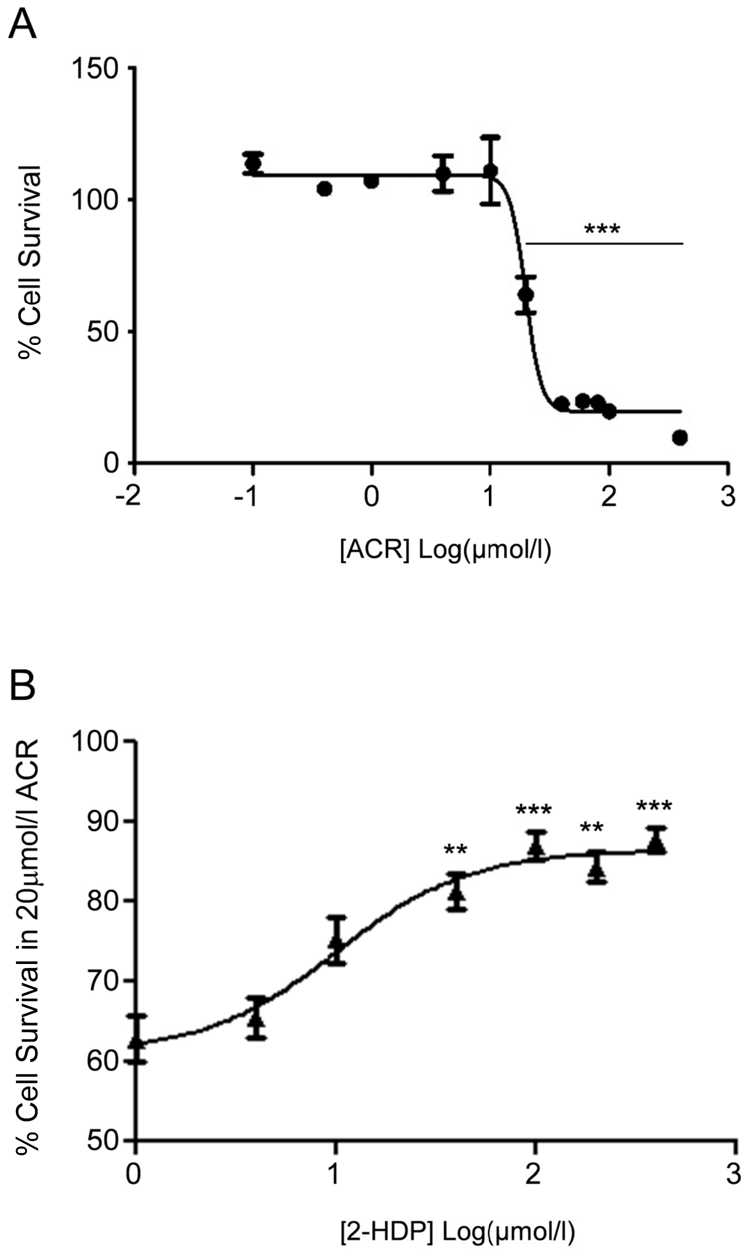

ESM Fig. 1 2-HDP protects against ACR-induced cell death in cultured retinal Müller glia. **1a**. Concentration-response curve showing the effects of ACR on Müller cell survival quantified using the Promega RealTime-Glo MT assay. Data are presented as a percentage of untreated control cells. IC50=20 μmol/l (****p*<0.001 v untreated cells). **1b**. Concentration-response curve demonstrating the inhibitory effect of 2-HDP on Müller cell death induced by 20 μmol/l ACR. Data are presented as a percentage of untreated control cells (***p*<0.01, ****p*<0.001 v ACR only treated cells).

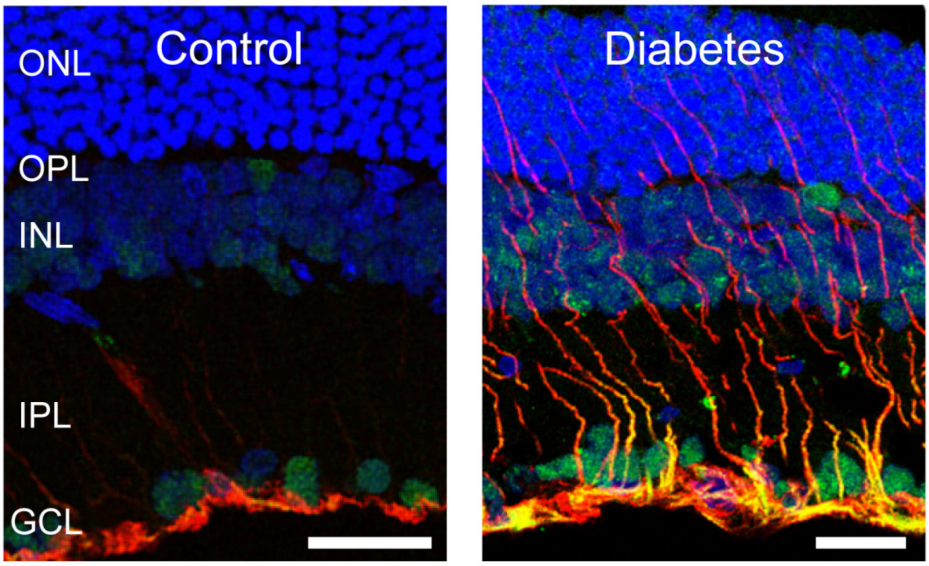

ESM Fig. 2 Confirmation of FDP-lysine accumulation in diabetic Müller glia. Retinal sections from control and diabetic rats (3-mo disease duration) were labelled with FDP-lysine (green), GFAP (red) and TO-PRO nuclear dye (1:1000; blue) and confocal images captured using a Leica SP5 confocal microscope. In control animals, GFAP expression was limited mainly to astrocytes within the ganglion cell (GCL) and lower, nerve fibre layers. In diabetes, the Müller cell end-feet and radial processes acquired strong GFAP immunoreactivity. Yellow staining indicates co-localisation of FDP-lysine and GFAP in the diabetic Müller cells. Scale bars = 50 μm
